# Evaluation of the quality of patient involvement in a patient-led analysis of the lived experience of a rare disease

**DOI:** 10.1186/s40900-023-00445-2

**Published:** 2023-05-25

**Authors:** Dawn Lobban, Jacqui Oliver, Kelly Davio, Kenza Seddik, Veronica Porkess

**Affiliations:** 1 Envision Pharma Group, Wilmslow, UK; 2 Patient Author, MG Patient Advocate, Richmond, London, UK; 3 UCB Pharma, Colombes, France; 4 UCB Pharma, Slough, UK

**Keywords:** Lived experience, Myasthenia gravis, Patient and public involvement, Patient author, Patient perspective, Qualitative research

## Abstract

**Background:**

Patients themselves are best placed to provide insights on the lived experience and to lead the analysis of such insights to bring the patient voice into peer-reviewed literature. In doing so, they can meet the authorship criteria for subsequent research publications. It is important to evaluate patient engagement to identify ways to improve future collaborations. Here, we describe the approach taken during a patient-led and patient co-authored analysis of the lived experience of generalized myasthenia gravis, which may be applicable to other indications. We also assessed the quality of patient engagement throughout the research project.

**Methods:**

We used self-reported experience surveys based on the Patient Focused Medicines Development Patient Engagement Quality Guidance criteria for assessing patient engagement. The surveys were adapted to focus on individual projects and assessed eight domains using a five-point Likert scale. In September 2020, we invited eight patient council members to complete a self-reported experience survey following qualitative lived experience data generation. We calculated the average experience score as a percentage of the maximum possible score. Patient authors (n = 1) and non-patient authors (n = 3) were invited to complete a similar survey in November 2021, with questions customized for relevance, to evaluate the authorship experience following publication of the research.

**Results:**

Overall, patient council members had a positive experience of taking part in this study, with an average experience score of 90% (71.6/80.0; n = 8). The patient author and non-patient authors rated their authorship experience highly, with average experience scores of 92% (78.0/85.0) and 97% (63.3/65.0), respectively. There were key aspects that contributed to the overall project success (e.g., ensuring that all participants were aligned on the project objectives at the outset and understood their roles and responsibilities). We also identified elements of the approach that could be improved in future collaborations.

**Conclusion:**

In this patient-led analysis, patient council members, patient authors and non-patient authors had a positive experience of being involved in the project. We gained useful insights into elements that contributed to the project’s success and ways to improve future patient-led projects on the lived experience.

**Supplementary Information:**

The online version contains supplementary material available at 10.1186/s40900-023-00445-2.

## Abstract (plain language summary)

**Why did we do this research?:**

Involving patients in research is becoming more common. Listening to patients can help healthcare teams to better understand the impact of living with a condition. Some patients taking part in research can go on to be an author of an article that describes the findings. We describe how we assessed the quality of patient involvement in a research project to better understand views of patients living with a rare condition.

**How did we assess patient involvement?:**

We asked patients to complete surveys to find out about their experience of taking part in a patient-led analysis of a rare condition. This can highlight challenges that they may have faced during the project.

**What were the main findings?:**

Patients who took part in this research and those who were authors of the research article had a positive experience. There were several key factors that helped the project to be a success. We also identified ways to improve future projects designed to gain patient insights on living with other conditions.

**Supplementary Information:**

The online version contains supplementary material available at 10.1186/s40900-023-00445-2.

## Background

The importance of involving patients throughout the drug development life cycle is increasingly recognized by multiple stakeholders, including patients [1, 2]. In particular, the benefits of partnering with patients to gain unique insights into their perspectives of living with a condition are well-recognized, especially in rare diseases [3, 4]. Patient-reported outcomes instruments provide useful information on the impact of living with a condition on people’s quality of life, but they do not provide the in-depth understanding gained from learning about the lived experience. Patient insights can help healthcare professionals better understand the unmet needs of patients, which may facilitate shared decision-making [5–7]. Importantly, seeking insights from patients themselves encourages patients to actively participate in all aspects of their condition, including advocacy, and draws attention to priorities for their own care [8]. Gaining insights can also empower patients in the knowledge that their lived experience is important and valuable.

Not only are patients themselves best placed to provide insights into their own condition, but they are also best placed to analyze and interpret insights from broader patient populations. Such patient-led research can in turn provide patients with an opportunity to meet the International Committee of Medical Journal Editors (ICMJE) criteria for authorship so they go on to author peer-reviewed publications describing the lived experience. However, there are perceived barriers to patient authorship. For example, a recent survey highlighted that only 3.6% of editors-in-chief (4 of 110) have policies regarding patient authorship [9]. Potential risks of including patients as authors have been identified (e.g., increased resource needs and opportunity costs, imbalance of power dynamics in the authorship group and risk of nonrepresentative insights from patient groups that are less diverse than the wider patient population) [10]. Despite these barriers, the number of patients authoring their research is increasing, driven by growing interest from patients themselves and recognition by major funders who support patient authorship [11].

Our patient-authored, peer-reviewed publication describes an analysis of the lived experience of a rare neuromuscular disease, myasthenia gravis (MG) [12]. MG is a rare, chronic and unpredictable autoimmune disease with a prevalence of around 1–2 per 10,000 people [13, 14]. Clinical symptoms of MG include muscle weakness and fatigue [15, 16], which can be restricted to certain muscle groups, such as the eyes (ocular myasthenia) or involve multiple muscle groups (generalized MG). Many people experience central fatigue (physical or mental) and may have comorbidities that impact their quality of life [15, 17, 18]. Importantly, people with MG may have symptoms that fluctuate over time so their symptoms may not always be evident to healthcare professionals or friends and family. Although there is a wide evidence base on the epidemiology and clinical symptoms of MG, further evidence is needed on the lived experience of MG from the patient’s own perspective.

In 2018, UCB Pharma established an international patient council to facilitate a patient-led approach to improving treatment for people with generalized MG. The patient council was a group of leading, national patient advocates with MG from Belgium, Finland, France, the Netherlands, Spain, the United Kingdom and the United States. Here, we describe the approach we used in the design and conduct of a patient-led and patient co-authored analysis of the lived experience of generalized MG, which may be applicable to other indications. We also describe the self-reported experience of being involved in the project either as a member of the patient council, as a patient author or non-patient author, to understand how this approach could be improved for all involved.

### Project approach for the published patient-led analysis

**Fig. 1:**
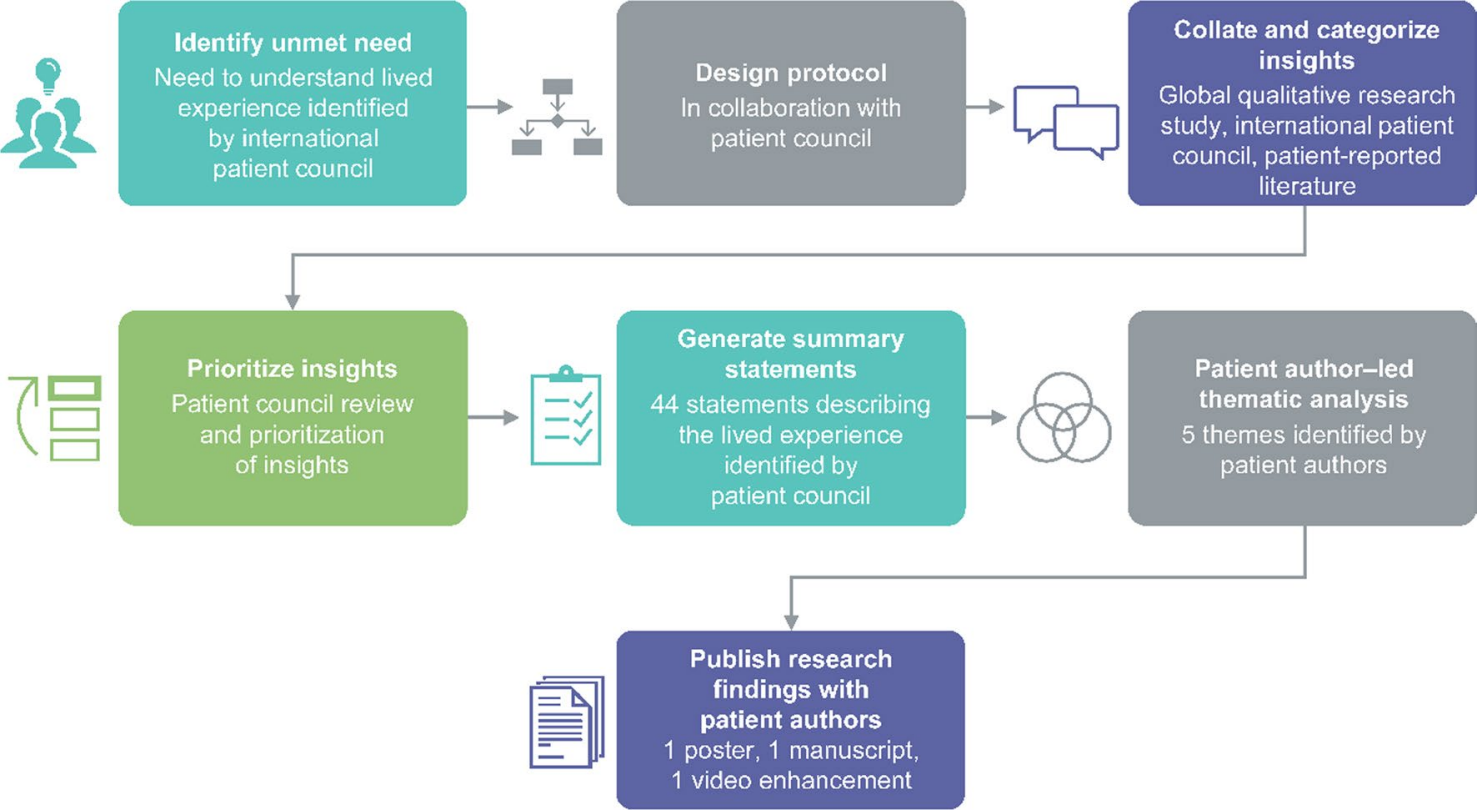
Project approach for insight identification, collation, prioritization and analysis

For this research, the patient council was composed of people with MG. Although family and caregiver views were considered, the focus was on those who have MG. We established a patient-led approach to the identification, collation, prioritization, analysis and publication of patient insights to describe the lived experience (Fig. 1). Care was taken to ensure that the proposed approach was discussed with all council members, and that they had an opportunity to input. Further details of the data sources used to extract insights and methods used in this study have been published elsewhere [12].

#### Identification of unmet needs

Patient council members highlighted a lack of understanding among many healthcare professionals about the lived experience of MG. They noted a lack of published patient-reported data and identified the need for a qualitative research analysis to better understand and report the patient perspective of living with MG.

#### Insight collation and categorization

At the start of the patient-led analysis, council members provided suggestions for domains that reflected different aspects of the lived experience of MG. These domains provided a framework representing the lived experience of MG and are described in full detail in the original patient-led analysis [12]. Patient insights into the lived experience of MG based on qualitative data reported in the literature and direct patient quotes were extracted from three sources (Table 1). Insights and quotes extracted from the data sources were categorized into nine descriptive domains: physical, psychological, social, activities and participation, reproduction and parenting, controlled/inadequately controlled, flare-ups and myasthenic crises, treatment burden and unmet needs. Two researchers (Dawn Lobban, Jennie Hepburn) systematically collated and categorized patient insights, with specific quotes noted to support the extracted insights. Researchers discussed and cross-checked extracted insights to ensure the source data were accurately represented and to minimize researcher interpretation.

**Table 1:** Information sources used to gain patient insights on the lived experience of MG

Source	
*Global qualitative research study* Qualitative research study of 54 people with MG or their carers from seven countries	Objective was to extract patient insights and quotes that focused on the ongoing management of people receiving treatment for MG Conducted by external researchers (Branding Science Ltd) on behalf of UCB Pharma using web-assisted telephone or in-home individual interviews
*Literature review* 32 peer-reviewed research publications, one newsletter and one book, that present patient-reported outcomes or experiences of living with MG	Researchers searched the peer-reviewed literature, predetermined patient and sociology journals and gray literature Two researchers screened articles for relevance to identify information relating to patient experience or patient-reported outcomes
*International MG patient council meeting report* Discussions among six council members living with MG	Detailed discussions among patient council members who lived with MG and were patient advocates in their local communities Extracted insights from the meeting report that were not already captured in the qualitative research study

*MG* myasthenia gravis

Further details of these data sources are provided in the Electronic Supplementary Material in the published patient-led analysis article [12]

#### Generation of summary statements and patient author–led thematic analysis

Insights were reviewed and prioritized by patient council members using an online survey. Patients were asked to consider how well each insight represented the lived experience of MG. Each council member chose five insights that they felt best represented each of the domains from a global view based on their experience as patient advocates, rather than their personal experience. Patient council members had in-depth discussions about the survey findings during two half-day virtual workshops in August 2020 and subsequently developed summary statements of the lived experience.

Patient authors finalized these statements after the meeting. Based on collective experience, the patient council identified 44 summary statements across nine domains that they considered best represented the lived experience of generalized MG. As it has been reported that people living with chronic diseases can experience some sense of personal growth [19], and to ensure balance in the data, patient council members were also asked to share any positive aspects of living with MG. Patient authors reviewed the statements in an online meeting (October 2020) to further summarize the statements into five overarching themes to best describe the patient perspective of living with MG.

#### Publishing the research findings

Two patient authors and three non-patient authors worked together to report the findings of the analysis. Although both patient authors did have some prior experience of publications, the company publication lead (Veronica Porkess) explained the publication process to them at the outset to ensure that they both understood their role, responsibilities and the time commitment associated with authorship of company-sponsored research publications. In addition, the publication lead provided plain language versions of some key documents: Good Publication Practice guidelines, author agreement form and confidentiality agreement [20]. Medical writing support was provided to support patient and non-patient authors to publish the research findings.

Initially, the data were submitted as an abstract and presented as an audio-enhanced poster at a large neurology conference in the United States [21]. The full manuscript was subsequently published in a leading neurology journal [12]. A short supporting video was developed to increase interest, reach and accessibility of the data [22]. The patient-led analysis of the lived experience of MG has received high online attention (6504 article accesses, 7 citations and an Altmetric score of 263 as of March 2023 since publication in October 2021). The article received mentions in 35 news outlets and was ranked 1st of the tracked articles of a similar age in Neurology and Therapy. The involvement of patient council members and patient co-authors in this project is summarized in Additional file 1 following the Guidance for Reporting Involvement of Patients and the Public (GRIPP2), which have been developed to improve the reporting of patient and public involvement in health research [23, 24].

### Assessing the self-reported experience of patient involvement in the patient-led analysis

We assessed the involvement of patient council members, patient authors and non-patient authors using a self-reported experience online survey (Additional file 2). The survey was based on the Patient Focused Medicines Development (PFMD) Patient Engagement Quality Guidance criteria for planning or assessing patient engagement activities [25]. Information on the survey and how it was developed has been reported previously [20, 26]. Questions on the results and outcomes of this project were also included in the survey based on recommendations in the PFMD Book of Good Practices and GRIPP2 [23, 24, 27].

Overall, eight domains were assessed using a five-point, psychometric Likert scale (strongly disagree to strongly agree) and an overall score was calculated as a percentage of the maximum possible score. Questions were customized for relevance (i.e., experience of being involved “as a member of the patient council” or “as a patient author”) and were reviewed and approved by target audience representatives. In September 2020, nine members of the patient council were invited to complete the patient experience survey comprising 16 questions to evaluate how well researchers worked with patient partners in the qualitative data generation. Eight members of the patient council completed the survey and one patient council member did not complete the survey, despite a follow-up email. Following the publication of the research findings, we gained feedback on the authorship experience from three non-patient authors (13 questions) and one patient author (17 questions) in December 2021. Following preparation of the lived experience manuscript, one of the patient authors sadly passed away so only one patient author completed the authorship experience survey.

**Fig. 2:**
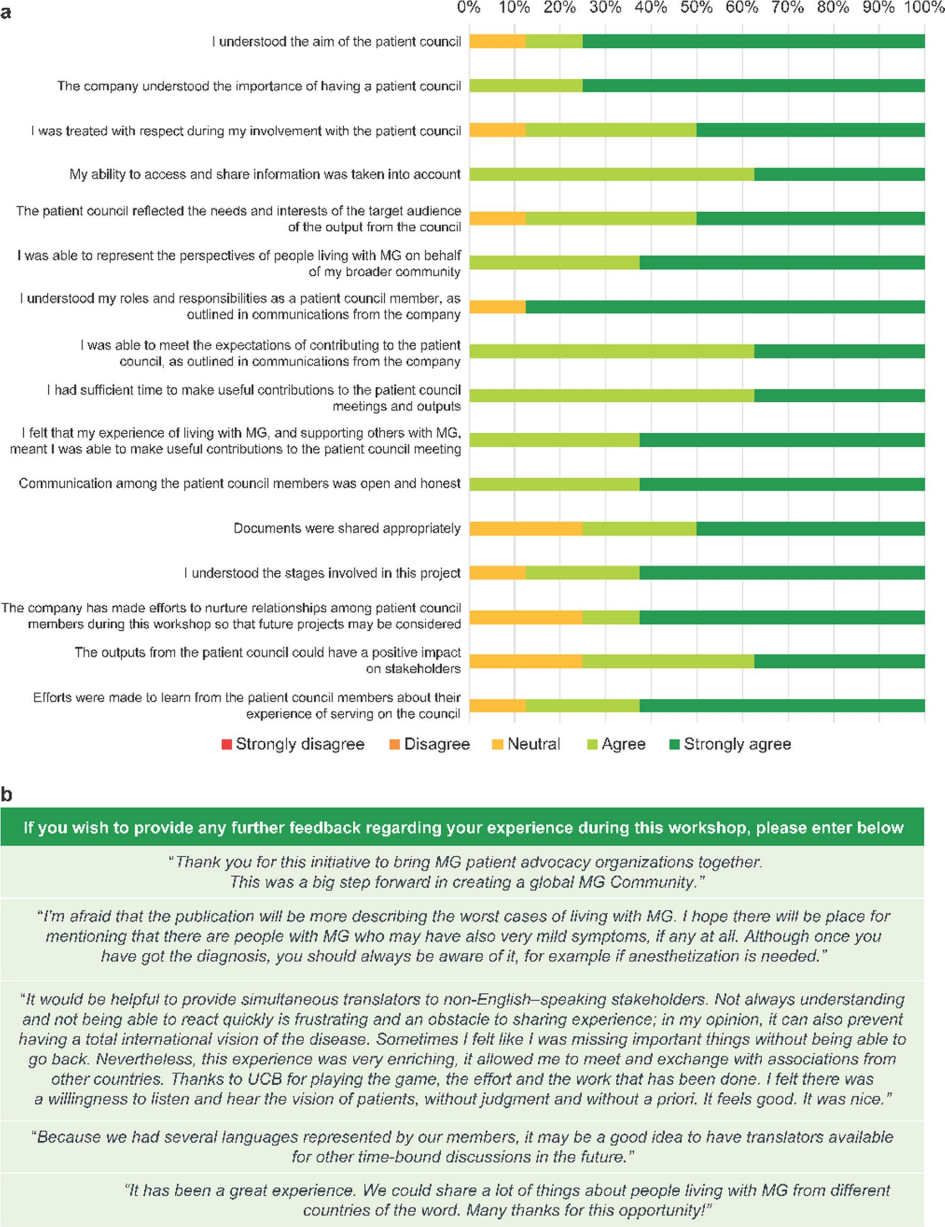
Patient council experience of involvement in qualitative data generation: **a** patient council members survey responses; **b** additional feedback from patient council members on their experience. MG, myasthenia gravis

**Fig. 3:**
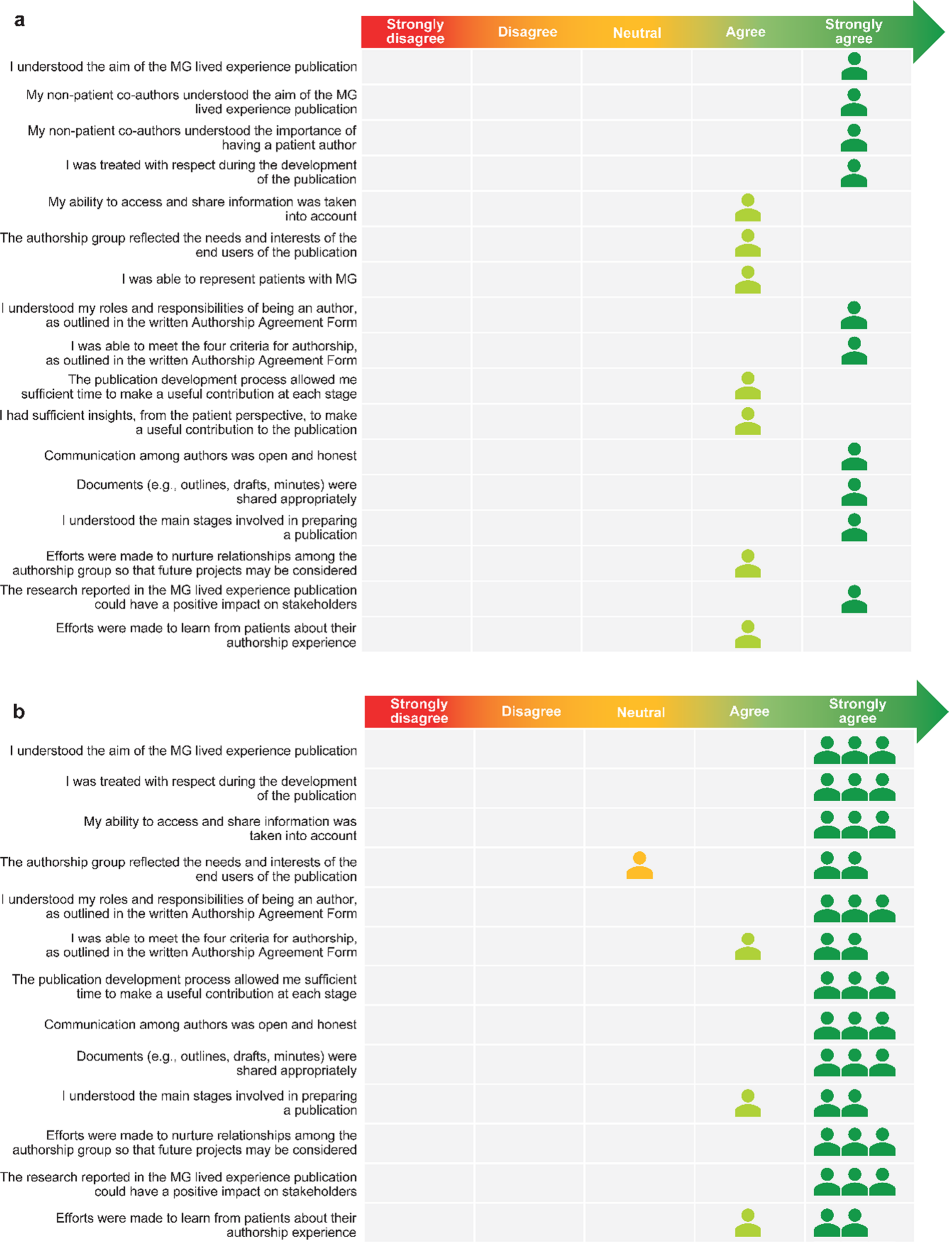
Self-reported experience of involvement in patient co-authored publication: **a** patient author survey responses; **b** non-patient author survey responses. MG, myasthenia gravis

Overall, patient council members rated their experience of taking part in this analysis as positive, with an average patient experience score of 90% (71.6/80.0; n = 8). The patient experience survey results from the patient council members are summarized in Fig. 2a. Patients provided additional open-text feedback on their experience of being involved in the analysis (Fig. 2b). Involvement in the patient co-authored publication was also rated highly: experience score was 92% (78/85; n = 1) for the patient author (Fig. 3a) and 97% (63.3/65; n = 3) for non-patient authors (Fig. 3b).

### Key learnings to optimize the approach

Based on our own experience of the approach and the feedback provided by the patient council members and authors, Table 2 captures key aspects that contributed to the overall success of the approach. We also reflect on elements of the approach that could be improved in future studies. Consensus on the key aspects leading to success and elements for improvement was achieved through a virtual discussion followed by two rounds of online written review.

**Table 2:** Key learnings to optimize the approach

*Elements that contributed to the overall success of the approach*
Identify and involve patient partners from the project outset to ensure valuable input into defining the unmet need, designing, analyzing and publishing the data
Ensure objectives and project approach, including timelines, are agreed by all participants at the start of the project and ensure everyone understands the project objectives and process as well as their own roles and responsibilities within it
Involve company compliance teams from the outset and throughout the project to ensure the successful and smooth delivery of the project, including local teams for each patient where required
Agree on potential patient co-authors at the start of the project to ensure they can go on to fulfill ICJME authorship criteria, and to manage expectations of any non-author participants
Take time to explain the publication process to patient co-authors and provide plain language versions of explanatory materials where appropriate (e.g., authorship requirements, publication process, confidentiality forms)
Provide patient advocates with sufficient time to review documents and literature
Organize meetings at times to suit patient advocates, not to fit in with the schedules of researchers or industry attendees
Consider the optimal online platform for discussions and demonstrate functionality in advance
Gather feedback on both the positive and negative aspects of living with the condition to ensure a balanced portrayal of the lived experience
Design the workshop agendas to optimize input and allow an experienced facilitator to capture feedback from all attendees
Share the meeting output/summary with all the participants and ensure they review the final version of the paper
Design the analysis to limit researcher bias and ensure the data reflect the feedback from the patient advocates
Consider publication extenders, such as short videos, to enhance the article
*Elements of the approach that could be improved*
Consider all potential ethics board approvals at the outset of any qualitative data generation, as journal requirements may differ from local market research guidance
Provide simultaneous translation as needed during workshop discussions to ensure that all patient advocates can follow and input despite their native language
Divide workshop sessions into manageable time allocations for the patients involved. This analysis involved two half-day workshops, but running more sessions of shorter duration may be more appropriate for the patient advocates
Be realistic about the overall project timelines. Patient advocates are often managing their condition and are juggling other priorities
Consider an effective and compliant dissemination plan, working with the publishers, to optimize the reach of the published data to multiple audiences

*ICMJE* International Committee of Medical Journal Editors

**Table:** 

The patient co-author’s perspective (Kelly Davio)	
This patient-led approach presented a unique opportunity for patient advocates from multiple countries to describe their lived experience (and their broader patient communities’ lived experiences) of MG. It also allowed the insights generated in this process to be documented in the peer-reviewed literature, which I believe will contribute to greater understanding of patients’ experiences, needs and perspectives. As an individual patient, I found the process of engaging with the patient council and of authorship to be a unique and indeed empowering opportunity to share and represent the views and needs of the MG community. I believe this approach can be successfully used among patients with other conditions to amplify the patient voice and to help to create a more nuanced understanding of patients’ perspectives and priorities among healthcare decision-makers	

## Conclusions

Patient involvement in research publications is a relatively new area. As recently highlighted, patient engagement is not always successful, e.g., there can be an unconscious bias towards patient partners and a lack of support to fully include patients [28]. As such, it is important to reflect on the patient experience of being involved to improve future patient involvement initiatives [28]. We gained useful insights into ways to improve patient involvement in future research and patient co-authored publications. By involving patients at the project outset, clearly outlining roles and responsibilities and being flexible about how patients provided input, we enabled patients to publish valuable insights into the lived experience of MG.

We used a patient-led approach to collate, prioritize and analyze insights from people living with MG. This provided a comprehensive view of the patient perspective of the lived experience of MG. Patient council members had extensive experience as patient advocates and had interacted with a broad range of patients from across the world. This enabled council members to identify wider insights that best represented the patient perspective of living with MG. These insights were published to help healthcare professionals better understand their patients’ experience of living with MG that may not be evident in routine clinical appointments. Importantly, this could facilitate understanding of the impact of symptoms (e.g., on a patient’s quality of life, mental health, relationships, participation in activities). This approach was also a way for patient advocates to raise their voice and ensure their insights were included in the peer-reviewed literature to improve awareness of living with MG for anyone with an interest in the disease.

Although the number of patient authors is currently low and difficult to measure [11, 30], evidence-based guidance and resources to facilitate patient authorship are emerging [10, 31–33]. This guidance can help to overcome the perceived barriers to patient authorship. The recent Good Publication Practice guidelines clearly recognize that patients can provide important input into publications, and patients who meet authorship criteria should be listed as authors [34]. For example, a systematic literature review co-authored by patients and non-patient authors explored the risks and benefits of involving patients in preparing health research peer-reviewed publications [10]. The review provides 21 evidence-based recommendations for involving patients before, during and after a manuscript is developed. To assist conversations between researchers and patient authors, members of the Strategy for Patient-Oriented Research Chronic Pain Network's Patient Engagement Committee have published guidance on patient authorship [31].

Practical open-access resources are now available to support patient authors and companies and academic collaborators working with patients [20, 35]. The self-directed online Patients in Publications course trains patient advocates on how to engage in publications as authors and peer-reviewers [35]. Plain language resources have been co-developed with patient advocates that explain how patients can meet the ICMJE authorship criteria, provide an overview of the publication process, outline the rights and responsibilities and provide guidance on author disclosures [20]. Although we know that patient authorship is increasing, currently it can be difficult and time-consuming to identify patient-authored publications [11, 30]. The affiliation search function in PubMed has been proposed as a quick and easy way to identify patient-authored publications, but consensus is needed for stakeholders on a suitable standard meta tag (such as Patient Author) or set of meta tags to track patient authors [11]. Improving our understanding of the true extent of patient authorship could encourage more patients to contribute their perspectives to the peer-reviewed literature.

Evaluating the experience of patients engaged in research can provide valuable feedback and identify areas to improve future collaborations. Patient experience tools allow patients and non-patients to reflect on their experience of being involved in a research project and can highlight challenges they may have faced as well as benefits of taking part in the research. Overall, in this project, patients involved as a patient council member or as a patient author had a positive experience. Since publication, the patient-led analysis of the lived experience of MG has received high online attention, demonstrating interest in this type of analysis to better understand the lived experience [12]. Whereas similar Altmetric scores may be seen for publications on common conditions, these scores are high for a publication on a rare disease. A limitation of this research is that only nine participants were involved in this research who were all patients living with MG. However, the results of this analysis may be informative for people affected by MG in the wider sense including “the family and those voluntarily caring for those with the medical condition(s), patient advocates, and patient groups” [25, 29]. This patient-led approach provides a framework that other researchers could follow in future research and adapt to gain valuable insights on the lived experience of other conditions.

## Supplementary Information

**Additional file 1**. Patient involvement reported using the GRIPP2 short form.

**Additional file 1**. Self-reported experience surveys.

## Data Availability

Not applicable.

## Abbreviation

GRIPP2: Guidance for Reporting Involvement of Patients and the Public
ICMJE: International Committee of Medical Journal Editors
MG: Myasthenia gravis
PFMD: Patient Focused Medicines Development
