# Supplementary material for: Evaluation of the quality of patient involvement in a patient-led analysis of the lived experience of a rare disease
Source: Res Involv Engagem. 2023 May 25;9:35. doi: 10.1186/s40900-023-00445-2 (PMC10214587; doi:10.1186/s40900-023-00445-2)
Supplement: Supplementary file 1 — Additional file 1. Patient involvement reported using the GRIPP2 short form. [file 40900_2023_445_MOESM1_ESM.docx]

**Supplementary information for Evaluation of the quality of patient involvement in a patient-led analysis of the lived experience of a rare disease**

**Additional file 1** Patient involvement reported using the GRIPP2 short form

| Section and topic | Item |
| --- | --- |
| 1. Aim | Report the aim of PPI in the study   - To collaborate with patient advocates to gain their insights on the lived experience of MG - To partner with patient authors to publish these insights to help healthcare professionals better understand the unmet needs of people living with MG |
| 1. Methods | Provide a clear description of the methods used for PPI in the study   - Members of the patient council were invited to take part in the analysis. They identified unmet needs, suggested domains for a framework representing the lived experience of MG, reviewed and prioritized insights. Patient council members participated in a virtual workshop to develop summary statements of the lived experience - Two patient council members were invited to co‑author the research findings and led the thematic analysis - Patient council members and authors completed self‑reported experience surveys to assess their involvement in the analysis or subsequent publication |
| 1. Study results | Outcomes – report the results of PPI in the study, including both positive and negative outcomes  Positive outcomes:   - Publishing the research findings addressed an unmet need identified by the patient council. The publication has generated high online attention highlighting the importance of understanding patients’ lived experience. One patient commented that they found the “experience enriching” - The patients involved in the study represented views of the wider MG community, as they were advocates in their local communities in Europe and United States - As HCPs were not involved, patients could speak freely about their perspective without feeling “less than” an HCP. The presence of a neurologist would have changed the process substantially   Negative outcomes:   - Interactions with patient advocates were primarily in English, which posed challenges for some members and may have limited their interaction |
| 1. Discussion and conclusions | Outcomes – comment on the extent to which PPI influenced the study overall. Describe positive and negative effects   - Patients were central to the research described in the analysis, providing insights into the negative and positive aspects of living with MG |
| 1. Reflections/critical perspective | Comment critically on the study, reflecting on the things that went well and those that did not, so others can learn from this experience   - Overall, this was a very successful analysis in which the patient council members played a key role – see Table 2 for further details |

GRIPP2, Guidance for Reporting Involvement of Patients and the Public; HCP, healthcare professional; MG, myasthenia gravis; PPI, patient and public involvement.
