# Supplementary material for: Evaluation of the quality of patient involvement in a patient-led analysis of the lived experience of a rare disease
Source: Res Involv Engagem. 2023 May 25;9:35. doi: 10.1186/s40900-023-00445-2 (PMC10214587; doi:10.1186/s40900-023-00445-2)
Supplement: Supplementary file 2 — Additional file 1. Self-reported experience surveys. [file 40900_2023_445_MOESM2_ESM.docx]

**Supplementary information for Evaluation of the quality of patient involvement in a patient-led analysis of the lived experience of a rare disease**

**Additional file 2** Self-reported experience surveys

1. **Patient Council experience survey**

| **Statement** | Strongly disagree | Disagree | Neutral | Agree | Strongly  agree |
| --- | --- | --- | --- | --- | --- |
| **Shared purpose** | | | | | |
| 1. I understood the aim of the Patient Council |  |  |  |  |  |
| 1. UCB Pharma understood the importance of having a Patient Council |  |  |  |  |  |
| **Respect and accessibility** | | | | | |
| 1. I was treated with respect during my involvement with the Patient Council |  |  |  |  |  |
| 1. My ability to access and share information was taken into account (e.g., ability to attend Patient Council meetings, time to review documents) |  |  |  |  |  |
| **Representativeness of stakeholders** | | | | | |
| 1. The Patient Council reflected the needs and interests of the target audience of the output from the Council (i.e., a publication describing the lived experience and unmet needs of people with MG intended for neurologists, people with MG ad others with an interest in MG) |  |  |  |  |  |
| 1. I was able to represent the perspectives of people living with MG on behalf of my broader community |  |  |  |  |  |
| **Roles and responsibilities** | | | | | |
| 1. I understood my roles and responsibilities as a Patient Council member, as outlined in communications from UCB Pharma |  |  |  |  |  |
| 1. I was able to meet the expectations of contributing to the Patient Council, as outlined in communications from UCB Pharma |  |  |  |  |  |
| **Capacity and capability for engagement** | | | | | |
| 1. I had sufficient time to make useful contributions to the Patient Council meetings and outputs |  |  |  |  |  |
| 1. I felt that my experience as a person living with MG, and supporting others with MG, meant I was able to make useful contributions to the Patient Council meetings |  |  |  |  |  |
| **Transparency in communication and documentation** | | | | | |
| 1. Communication among the Patient Council members was open and honest |  |  |  |  |  |
| 1. Documents (e.g., invitations, agendas, pre‑reading material, meeting summaries) were shared appropriately |  |  |  |  |  |
| **Continuity and sustainability** | | | | | |
| 1. I understood the stages involved in this project |  |  |  |  |  |
| 1. UCB Pharma has made efforts to nurture relationships among Patient Council members during this particular workshop so that future projects may be considered |  |  |  |  |  |
| **Results and outcomes** | | | | | |
| 1. The outputs from the Patient Council could have a positive impact on stakeholders (e.g., people with MG, healthcare teams, researchers) |  |  |  |  |  |
| 1. Efforts were made to learn from the Patient Council members about their experience of serving on the council |  |  |  |  |  |

1. **Authorship experience survey**

| **Statement** | Strongly disagree | Disagree | Neutral | Agree | Strongly  agree |
| --- | --- | --- | --- | --- | --- |
| **Shared purpose** | | | | | |
| 1. I understood the aim of the MG lived experience publication |  |  |  |  |  |
| 1. My non-patient co-authors understood the aim of the MG lived experience publication (note: please only answer this question if you are a patient author) |  |  |  |  |  |
| 1. My non-patient co-authors understood the importance of having a patient author (note: please only answer this question if you are a patient author) |  |  |  |  |  |
| **Respect and accessibility** | | | | | |
| 1. I was treated with respect during the development of the publication |  |  |  |  |  |
| 1. My ability to access and share information was taken into account (eg, ability to attend author meetings, time to review documents) |  |  |  |  |  |
| **Representativeness of stakeholders** | | | | | |
| 1. The authorship group reflected the needs and interests of the end users of the publication (eg, neurologists, people with MG, other healthcare team members) |  |  |  |  |  |
| 1. I was able to represent patients with myasthenia gravis (note: please only answer if you are a patient author) |  |  |  |  |  |
| **Roles and responsibilities** | | | | | |
| 1. I understood my roles and responsibilities of being an author, as outlined in the written Authorship Agreement Form |  |  |  |  |  |
| 1. I was able to meet the four criteria for authorship, as outlined in the written Authorship Agreement Form:- Provide a valuable contribution to the publication -Provide useful comments at each stage of writing the publication - Read and approve the final version of the report - Be willing to take responsibility for the accuracy and integrity of the publication, and help to answer questions about the publication |  |  |  |  |  |
| **Capacity and capability for engagement** | | | | | |
| 1. The publication development process allowed me sufficient time to make a useful contribution at each stage |  |  |  |  |  |
| 1. I had sufficient insights, from the patient perspective, to make a useful contribution to the publication (note: please only answer if you are a patient author) |  |  |  |  |  |
| **Transparency in communication and documentation** | | | | | |
| 1. Communication among authors was open and honest |  |  |  |  |  |
| 1. Documents (eg, outlines, drafts, minutes) were shared appropriately |  |  |  |  |  |
| **Continuity and sustainability** | | | | | |
| 1. I understood the main stages involved in preparing a publication |  |  |  |  |  |
| 1. Efforts were made to nurture relationships among the authorship group so that future projects may be considered |  |  |  |  |  |
| **Results and outcomes** | | | | | |
| 1. The research reported in the MG lived experience publication could have a positive impact on stakeholders (eg, patients, researchers, healthcare team members) |  |  |  |  |  |
| 1. Efforts were made to learn from patients about their authorship experience |  |  |  |  |  |

MG, myasthenia gravis.

Further information on the Patient experience surveys is available in references [20, 26].
